# Foliar Nourishment with Nano-Selenium Dioxide Promotes Physiology, Biochemistry, Antioxidant Defenses, and Salt Tolerance in *Phaseolus vulgaris*

**DOI:** 10.3390/plants10061189

**Published:** 2021-06-11

**Authors:** Mostafa M. Rady, El-Sayed M. Desoky, Safia M. Ahmed, Ali A. Majrashi, Esmat F. Ali, Safaa M. A. I. Arnaout, Eman Selem

**Affiliations:** 1 Botany Department, Faculty of Agriculture, Fayoum University, Fayoum 63514, Egypt; sma20@fayoum.edu.eg; 2 Botany Department, Faculty of Agriculture, Zagazig University, Zagazig 44519, Egypt; sayed1981@zu.edu.eg (E.-S.M.D.); sea_sound16@zu.edu.eg (S.M.A.I.A.); 3 Department of Biology, College of Science, Taif University, P.O. Box 11099, Taif 21944, Saudi Arabia; aa.majrashi@tu.edu.sa (A.A.M.); a.esmat@tu.edu.sa (E.F.A.); 4 Botany and Microbiology Department, Faculty of Science, Zagazig University, Zagazig 44519, Egypt; eman8_extra8@yahoo.com

**Keywords:** soil salinity, nano-selenium, beans, anatomy, physiology, antioxidant enzyme

## Abstract

Novel strategic green approaches are urgently needed to raise the performance of plants subjected to stress. Two field-level experimental attempts were implemented during two (2019 and 2020) growing seasons to study the possible effects of exogenous nourishment with selenium dioxide nanoparticles (Se-NPs) on growth, physio-biochemical ingredients, antioxidant defenses, and yield of *Phaseolus vulgaris* (L.) plant growing on a salt-affected soil (EC = 7.55–7.61 dS m^−1^). At 20, 30, and 40 days from seeding, three foliar sprays were applied to plants with Se-NPs at a rate of 0.5, 1.0, or 1.5 mM. The experimental design was accomplished in randomized complete plots. The data indicate noteworthy elevations in indicators related to growth and yield; pigments related to effective photosynthesis, osmoprotectant (free proline and soluble sugars), nutrient and Se contents, K^+^/Na^+^ ratio, cell integrity (water content and stability of membranes), all enzyme activities; and all features related to leaf anatomy induced by Se-NPs foliar spray. Conversely, marked lowering in markers of Na^+^ content-induced oxidative stress (superoxide radical and hydrogen peroxide) and their outcomes in terms of ionic leakage and malondialdehyde were reported by foliar nourishment with Se-NPs compared to spraying leaves with water as an implemented control. The best results were recorded with Se-NPs applied at 1.0 mM, which mitigated the negative effects of soil salinity (control results). Therefore, the outcomes of this successful study recommend the use of Se-NPs at a rate of 1.0 mM as a foliar spray to grow common beans on saline soils with EC up to 7.55–7.61 dS m^−1^.

## 1. Introduction

Common bean (*Phaseolus vulgaris* L.) is an important legume crop for human food due to its wide range of production of seeds rich in protein content [1,2,3]. It is classified as a sensitive crop to salt stress [4]. It is produced under soil salinity conditions in Latin America (approximately 5–10%) and the Middle East (approximately 20–30%) [5].

Salinity stress is a major issue of concern for workers in the agricultural sector. It negatively influences crop production directly or indirectly. Many plants are vulnerable to salinity effects and are unequal to withstand low levels of salinity [2]. Irrigation with water of poor quality and/or lack of quantity is one of the key factors that accumulate salts in the soil, which discourages plant metabolism and physical and chemical process, and, consequently, plant growth and productivity [6]. Salinity reduces the chance of plant roots absorbing enough water due to the physiological drought stimulated by salinity, which limits the plants’ growth and productivity performance by inhibiting the metabolism process [7,8]. Plant metabolism is discouraged with salinity due to osmotic stress and Na^+^ and Cl^−^ ions toxicity, which contribute to the inhibition of plant growth, various physio-biochemical attributes, and outputs by the overproduction of some species of reactive oxygen (ROS): O_2_^•–^, H_2_O_2_, and OH^−^ [7,8,9,10]. Physiological drought, toxic influences of undesirable ions (Na^+^ and Cl^−^), and nutrient imbalance are key adverse effects that occur to plants growing on salt-affected soil [6,11]. Salt accumulations in leafy apoplasm to toxic scale lead to loss of cell turgor and cell shrinkage, leading to cell death. The physiological process most affected under salt stress is photosynthesis. This process is associated with a marked reduction in the chlorophyll content and closing of stomata, and thus the efficiency of photosynthesis [6,12,13].

Because of the aforementioned negative salt influences, it is important to use applicable approaches to enhance salinity tolerance and alleviate the deleterious influences of salinity stress [14]. Exogenous use of organic or inorganic substances is used to boost the productivity of common beans under salinity stress [15]. Selenium (Se) is a microelement essential for plant performance at low concentration, but it is toxic in high concentration for plants, and thus humans, animals, and microorganisms [16]. With a higher concentration (more than 100 mg per kg in plant tissues), Se toxicity is induced by the substitution of sulfur (S) with Se in amino acids, resulting in the production of nonfunctional proteins and enzymes. However, at lower concentrations (up to 16 mg per L), Se can boost plant tolerability, delay senescence, suppress oxidative stress, and promote aging seedling growth [17,18].

Recently, nanomaterials have become a desirable solution to many technological and environmental challenges in numerous fields [19]. Taking into account nanotechnology, agricultural sustainability, and ecological issues, it is imperative to examine the various approaches in which nanoproducts may alter plant metabolism associated with growth and development. At present, selenium nanoparticles (Se-NPs) have been introduced as highly stable nanoparticles to be used in the medical industry and as fertilizers in the agricultural and food industries [20]. Compared to selenates or selenites, Se-NPs have lower toxicity and higher bioactivity [21]. Se-NPs have been used as a foliar application recently to protect plants subjected to severe stress through improved antioxidant defense mechanisms [21], photosynthetic indices, and secondary metabolism [22]; however, there is still a long way to go to explain the effects of exogenous Se-NPs supplies on these traits in plants affected by salinity. Research on the performance, mechanism, and effect of nanoparticles such as Se-NPs in plants, as well as agricultural application, is still in the rudimentary stage.

Based on the foregoing, the present study aimed to define the fruitful functions of the exogenous application of Se-NPs in improving salt tolerance in salt-affected common bean plants by exploring the potential enhancing impacts of Se-NPs on plant performance in terms of growth and yield traits, as well as plant physiology and biochemistry.

## 2. Materials and Methods

### 2.1. Experimental Layout and Growing Conditions

The two seasons 2019 and 2020 were specified to conduct two field trials on a special farm at El-Noubaria (31.15651, 29.86418)**,** Egypt. For each season, planting began on February 25 and May 10 was the end time for the experiments. This period (25 February to 10 May) of the 2019 and 2020 seasons did not have any rainy days. Before starting each trial, soil samples were taken from the experimental farm site for analyzing the physicochemical and fertility status using the procedures described in [23,24], and the obtained data are shown in Table 1. Based on these analyses, the site’s soil had an EC_e_ value of 7.55–7.61 dS m^−^^1^ in soil paste extract for both seasons (Table 1), which classified the experimental soil as saline, as stated in [25].

As the most salt-sensitive *Phaseolus vulgaris* (L.) in Egypt, Bronco was identified for this study. The Egyptian Agricultural Research Center was the source from which healthy seeds of similar size, color, and disease-free were obtained. The seed surface was sterilized by rinsing in NaClO for 10 min, washed well with distilled water (d.W), and dried in the open air for 2 h. Sowing was implemented in late February for both trial seasons at 95 kg per ha to reach the seeding rate recommended for the commercial production of this crop. The seeds were grown on 10.5 m^2^ plots (long = 3.0 m × width = 3.50 m) in hills (4 seeds per hill, and 15–20 cm between every two hills) in rows spaced 60 cm apart. Immediately before the first watering, two healthy, strong seedlings were kept in each hill. Prior to field planting, 280 kg ha^−^^1^ (NH_4_)_2_SO_4_ (20% N) and 350 kg ha^−^^1^ Ca^2+^-superphosphate (15.5% P_2_O_5_) were added to the soil.

All treatment plots were organized in the open conditions of the selected special farm and the transplants/plants were preserved under natural climatic conditions (e.g., the mean temperatures and relative humidity were 31 ± 3 °C/17 ± 3 °C for day/night (approximately 12 h for each) and 62–66%, respectively). One level of foliar d.W was accomplished as a control, and there were three levels of Se-NPs (0.5, 1.0, and 1.5 mM) with three replicates per treatment. The experimental diagrams of all treatments were organized into a completely randomized design (CRD).

### 2.2. Foliar Application of Nano-Selenium (Se-NPs)

The Se-NPs was purchased from Sigma Aldrich Co. (Sigma-Aldrich, 99.5% purity of Se-NPs) with properties shown in Table 2.

When the plants reached 25 days old, they were sprayed with d.W (as a control), 0.5, 1.0, or 1.5 mM Se-NPs according to the treatments designed for this study. The spraying of d.W and the different concentrations of Se-NPs was repeated twice (i.e., the second and third sprays) when the plants were 35 and 45 days old, respectively. The concentrations of Se-NPs were selected based on our preliminary pot study, in which 11 concentrations (e.g., 0, 0.5, 1.0, 1.5, 2.0, 2.5, 3.0, 3.5, 4.0, 4.5, and 5.0) were examined. The concentrations 0.5, 1.0, and 1.5 conferred the best results (data not shown), and therefore they were selected for this main field study. Using a pressurized spray bottle, plants were foliar sprayed to run off. The volume sprayed of each solution was 25, 30, and 40 mL per plant at the three ages (25, 35, and 45 days old, respectively) with 5, 6, and 8 L of spray solution per 10.5 m^2^ plot considering 200 plants for the three ages, respectively. An appropriate surfactant (e.g., few drops of Tween-20) was applied to the spraying solutions.

### 2.3. Sampling Date and Sample Preparation for Different Determinations

After 8 weeks of planting, 9 plants were harvested indiscriminately from the middle rows in each plot (10.5 m^2^) of each of the four treatments in both seasons (2019 and 2020). After cleaning plant shoots using a water-filled bucket to remove any adhering dusts, the indices of plant growth, physiology, biochemistry, oxidative stress, and enzymatic activities, as well as leaf anatomy (measured only in the 2020 season), were assessed. In the merchantable green pod’s stage (10–11 weeks after planting), pods were reaped from 50 indiscriminately selected plants from the two outer rows of each trial plot to assess the indices of green yield. Data are displayed as an average of nine plants as three replicates.

### 2.4. Assessment of Growth and Green Yield Traits

After separating plants, shoots were subjected for taking lengths of shoots (cm), leaf number on each plant, area of leaves of each plant (cm^2^), and shoot dry weight (DW-Sh; g). DW-Sh was assessed after drying at 70 °C until two or three constant weights. Pods number for each plant and yield of green pods per ha (ton) were recorded.

### 2.5. Evaluation of Indicators of Plant Physiology and Biochemistry

Fresh tissue of a fully extended upper leaf devoid of the midribs was extracted using acetone (80%, *v*/*v*) to determine chlorophylls (a and b) and carotenoids [26]. The resulting supernatants were subjected to the monitoring of their optical densities (at wavelengths of 480, 645, and 663 nm) on a spectrophotometer apparatus.

Utilizing the same leafy material, the rate of both the transpiration (*Tr*) and net photosynthesis (*Pn*) of the photosynthetic system was detected using a portable photosynthesis system (LF6400XTR, LI-COR, USA). During 09:00–11:00 a.m., all measurements were recorded.

The methods described in [9,27,28,29,30,31,32,33] were utilized to determine each of proline, total soluble sugars, hydrogen peroxide (H_2_O_2_), superoxide (O_2_^•−^), peroxidation of membrane lipid level (evaluated as malondialdehyde level (MDA)), leakage of ions (EL), stability index of membranes (MSI), and relative content of tissue water (RWC), using a fully extended upper leaf devoid of the midribs.

Using a fully extended upper leaf devoid of the midribs, a constant number of 20 discs was specified to assess the total inorganic ions escaping from the leaves. EC1 (electrical conductivity) was recorded in the solution of the discs before heating. EC2 was recorded after heating at 45–55 °C for 0.5 h. Then, EC3 was recorded after boiling for 10 min. EL percentage was obtained using the following equation:

EL (%) = [(EC2 − EC1)/EC3] × 100(1)

Using a fully extended upper leaf devoid of the midribs, pieces with a constant weight of 0.2 g were used to determine the MSI. EC1 was recorded after heating the solution of 0.2 g sample on 40 °C for 0.5 h. EC2 was recorded after boiling the solution of another sample for 10 min. By applying the following equation, MSI percentage was obtained:

MSI (%) = [1 − (EC1/EC2)] × 100(2)

Using a fully extended upper leaf devoid of the midribs, a constant number of 2 cm diameter discs was specified to evaluate RWC. Immediately, fresh mass was recorded after the discs were weighed. Then, in the dark, the discs were water-saturated for an entire day. After gently removing the adhering water, the recording of the turgid mass was accomplished. After drying (at 70 °C until constant weights), the discs dry mass was taken. RWC (%) was recorded using this equation:

RWC (%) = [(fresh mass – dry mass)/(turgid mass − dry mass)] × 100(3)

To analyze nutrient (N, P, and K^+^) and Na^+^ contents, fully extended upper leaf samples were dried at 70 °C until reaching constant weights. After grinding, a 1:3 mixture of perchloric and nitric acids, respectively, was applied to digest the dried samples. Total N was determined by applying the micro-Kjeldahl method [34]. Total phosphorus was determined by colormetrically applying the ascorbic acid method [35]. The same solution was applied to determine K^+^ and Na^+^ ion contents using atomic absorption spectrophotometry [36].

### 2.6. Determination of Enzyme Activities

Using a fully extended upper leaf devoid of the midribs (0.5 g), the enzymatic extract was prepared and used as the supernatant obtained from the centrifugation (12,000× *g*, 4 °C, 0.25 h) of the leafy homogenate to assay enzyme activities (unit: mg^−1^ protein). The methods in [37,38,39,40] were utilized to assay catalase, peroxidase, ascorbate peroxidase, and glutathione reductase activities, respectively.

A frozen sample (500 mg) was homogenized in a mortar and pestle fixed on ice. The solution of homogenization was HEPES buffer (10 mL, 50 mM) and 0.l mM Na_2_EDTA (pH 7.6). After performing centrifugation (15,000× *g* for 15 min at 4 °C) to obtain the resulting homogenates, the resulting extract was applied to assay the concentration of protein and the activity of superoxide dismutase (SOD; unit, mg^−1^ protein). Overnight, the extract was dialyzed against a dilute homogenizing solution to separate the low-molecular-mass substances that interfere in the SOD assay. The protein–dye binding procedure [41] was used to measure the concentration of soluble protein. The procedure of Yu and Rengel [42] was used for the SOD activity assay. This procedure is established on the basis of observation of photochemical reduction inhibition of NBT (nitro blue tetrazolium). To assay total SOD, a 5 mL reaction mixture of 50 mM, 0.l mM, 50 mM, 13 mM, 0.025% (*w*/*v*), 75 µM, 2 µM, 0.2 mL for HEPES (pH 7.6), EDTA, Na_2_CO_3_ (pH 10.4), methionine, Triton X-l00, NBT, riboflavin, and enzyme extract, was prepared. Using a light intensity of 350 µM m^−2^ s^−1^, the mixture was illuminated for 15 min. One SOD activity unit was specified as an enzyme quantity that causes a 50% NBT reduction inhibition (observed spectrophotometrically on 560 nm).

### 2.7. Leaf Anatomy

Leaf specimens were secured from the middle internode with its leaf blade. The selected specimens were chosen from plants at the flowering stage for killing and fixing for 48 h in 100 mL of F.A.A. solution containing 50 mL of C_2_H_5_OH (95%), 5 mL of glacial acetic acid, and 10 mL of formalin, in addition to 35 mL of distilled water. Then, samples were exposed to washing using C_2_H_5_OH (50%). Dehydration and clearance were then performed with normal butyl alcohol series, and the resulting samples were embedded in paraffin wax (54–56 °C m.p). A rotary microtome was functioned to cut samples for 20 μm thick cross-sections that adhered (Haupt’s adhesive). The samples were then stained [43,44]. Slide photography was performed and then read with a light microscope equipped with a digital camera (canon power shot S80) connected to computer; the photographs were taken by zoom browsers ex. program. Sections were examined to detected histological manifestations of the chosen treatments and photomicrographed. The obtained leaf anatomical features were expressed in µm (x = 200 µm).

### 2.8. Analysis of the Resulting Data

Differences among all the resulting data means were compared at *p* ≤ 0.05 by the Duncan multiple range test. Analyses were performed, statistically, by applying COSTAT software (version 6.303, Berkeley, CA, USA).

## 3. Results

### 3.1. Growth, Productivity, and Photosynthetic Efficiency Responses of Salt-Stressed Common Bean to Foliar Nourishment with Se-NPs

The data presented in Table 3 and Table 4 indicate the lowest common bean plant growth (number and area of leaves per plant and length and dry weight of shoot per plant), yield (number of pods per plant and yield of green pods per hectare), and photosynthetic efficiency (leaf pigments (chlorophylls and carotenoids) and rate of both net photosynthesis (*Pn*) and transpiration (*Tr*)) as shown in the treatment of comparison (control, foliar spray with distilled water (d.W)), which reflected the negative effect of soil salinity. However, foliar nourishment with Se-NPs positively affected plant performances, as it led to significant elevations in the indices related to plant growth, productivity, and efficiency of photosynthesis comparing with the comparison treatment. The best-applied rate for Se-NPs was 1 mM, which maximized the results of all the growth and yield indices mentioned above. The same outcome trends were gained over both seasons (2019 and 2020).

### 3.2. Plant Tissue Cell Integrity Response of Salt-Stressed Common Bean to Foliar Nourishment with Se-NPs

**Figure 1:**
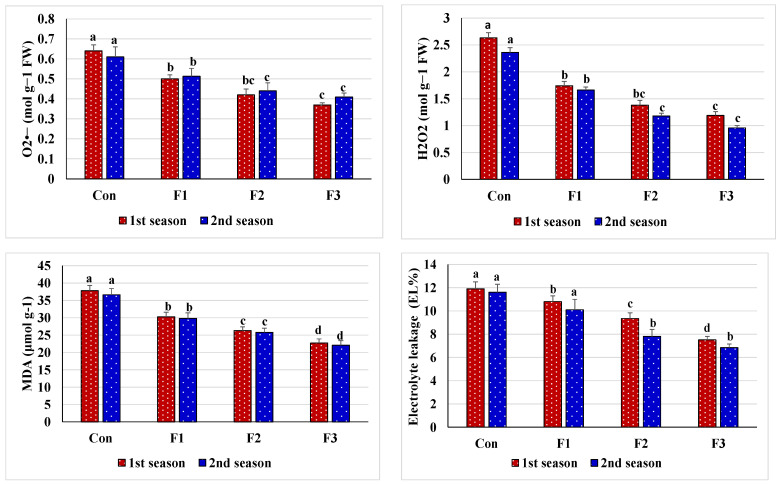
Oxidative stress markers and their outcomes responses of salt-stressed common bean plants (soil EC = 7.55–7.61 dS m^−1^) to foliar nourishment with selenium dioxide nanoparticles (Se-NPs) in two consecutive seasons. Above bars, different letters are considered significantly different at *p* ≤ 0.05. Con, control (spraying leaves with distilled water); F1, spraying leaves with 0.5 mM Se-NPs; F2, spraying leaves with 1 mM Se-NPs; F3, spraying leaves with 1.5 mM Se-NPs; MDA, lipid peroxidation measured as malondialdehyde; H_2_O_2_, hydrogen peroxide; O_2_^•−^, superoxide radical.

The comparison treatment, indicating salt stress and spraying leaves with d.W, presented adverse effects of salt stress such as reduced relative content of water (RWC), stability index of cell membranes (MSI), and contents of free proline, soluble sugars, and endogenous Se and raised levels of markers of oxidative stress (O_2_^•^^−^ and H_2_O_2_), leakage of electrolytes (EL), and peroxidation of membrane lipids (MDA) (Table 5 and Figure 1). However, leafy nourishment with Se-NPs positively modified these tested parameters of tissue cell integrity, as it resulted in significant promotion of RWC, MSI, free proline, soluble sugars, and Se contents due to minimized levels of oxidative stress markers, EL, and MDA comparing with the comparison treatment. The best-applied rate for Se-NPs was 1 mM, which preserved plant tissue cell integrity and increased endogenous Se to the desired optimal content. Both seasons (2019 and 2020) had the same outcome trends.

### 3.3. Enzyme Activity and Nutrient Content Responses of Salt-Stressed Common Bean to Foliar Nourishment with Se-NPs

**Figure 2:**
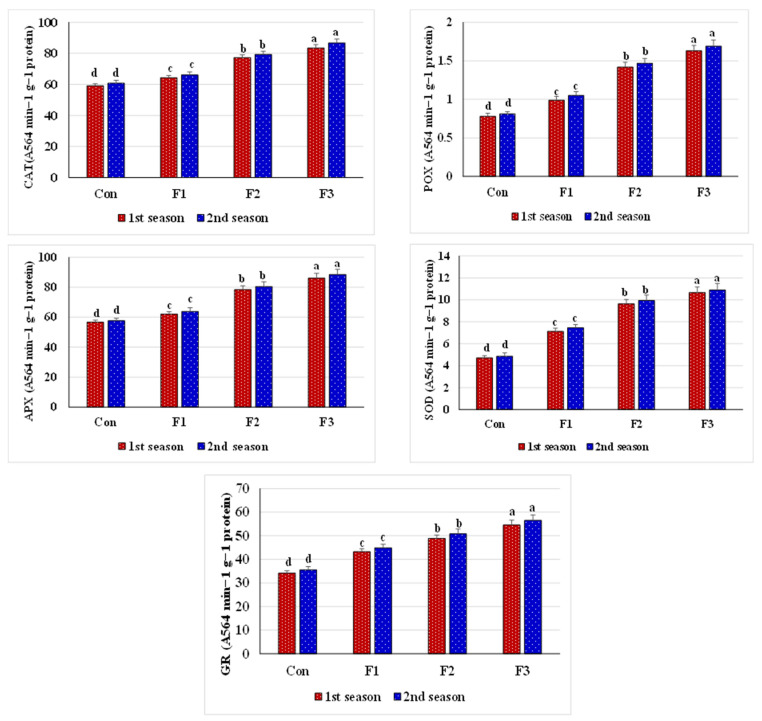
Enzyme activity response of salt-stressed common bean plants (soil EC = 7.55–7.61 dS m^−1^) to foliar nourishment with selenium dioxide nanoparticles (Se-NPs) in two consecutive seasons. Above bars, different letters are considered significantly different at *p* ≤ 0.05. Con, control (spraying leaves with distilled water); F1, spraying leaves with 0.5 mM Se-NPs; F2, spraying leaves with 1 mM Se-NPs; F3, spraying leaves with 1.5 mM Se-NPs; CAT, catalase; APX, ascorbate peroxidase; GR, glutathione reductase; SOD, superoxide dismutase; POX, peroxidase.

**Figure 3:**
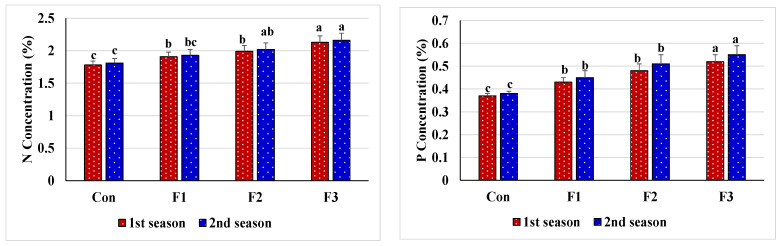

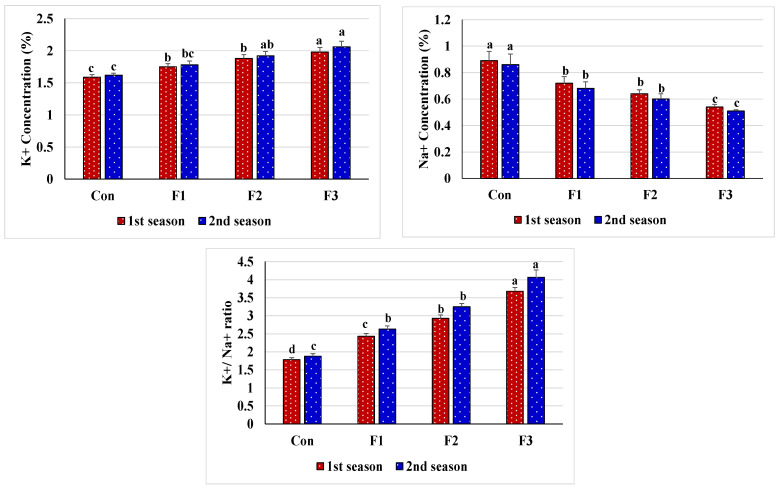
Nutrient content response of salt-stressed common bean plants (soil EC = 7.55–7.61 dS m^−1^) to foliar nourishment with selenium dioxide nanoparticles (Se-NPs) in two consecutive seasons. Above bars, different letters are considered significantly different at *p* ≤ 0.05. Con, control (spraying leaves with distilled water); F1, spraying leaves with 0.5 mM Se-NPs; F2, spraying leaves with 1 mM Se-NPs; F3, spraying leaves with 1.5 mM Se-NPs; N, nitrogen; P, phosphorus; K^+^, potassium ion; Na^+^, sodium ion.

Salt stress and spraying leaves with d.W (the comparison treatment) displayed the lowest activity of CAT, POX, APX, SOD, and GR; the lowest content of N, P, and K^+^; the lowest ratio of K^+^/Na^+^; and increased Na^+^ content (Figure 2 and Figure 3). However, foliar feeding with Se-NPs positively modified the tested enzyme activity and nutrient content, as this greatly improved all of the above parameters along with a considerable reduction in the Na^+^ content and a considerable rise in the ratio of K^+^/Na^+^ compared to the comparison treatment. The best-applied rate for Se-NPs was 1 mM, which sustained the plant antioxidative system and nutrient homeostasis. Both seasons (2019 and 2020) had the same outcome trends.

### 3.4. Anatomical Features Responses of Salt-Stressed Common Bean Leaf to Foliar Nourishment with Se-NPs

**Figure 4:**
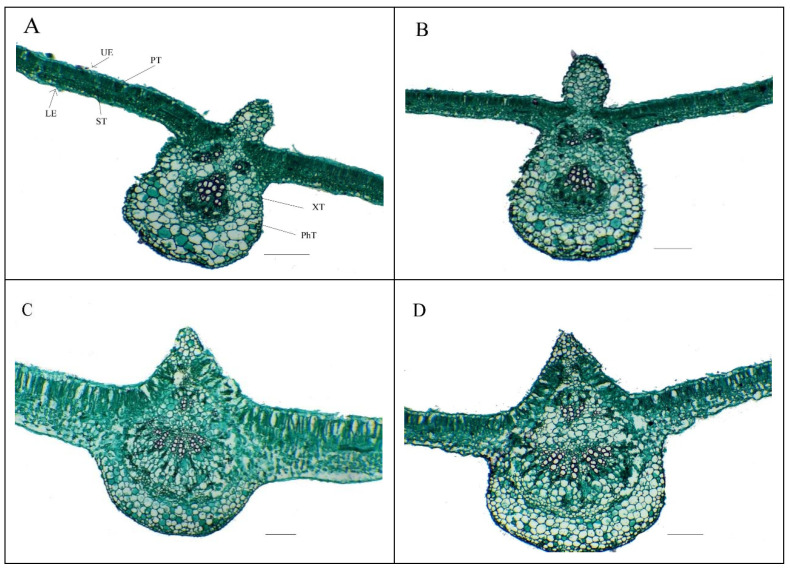
Leaf anatomy (transverse section through the leaflet blade) responses of salt-stressed common bean plants (soil EC = 7.55–7.61 dS m^−1^) to foliar nourishment with selenium dioxide nanoparticles (Se-NPs) in 2020 (second) season: (**A**) control (spraying leaves with distilled water); (**B**) spraying leaves with 0.5 mM Se-NPs; (**C**) spraying leaves with 1 mM Se-NPs; and (**D**) spraying leaves with 1.5 mM Se-NPs. UE, upper epidermis; LE, lower epidermis; PT, palisade tissue; ST, spongy tissue; XT, xylem tissue; PhT, phloem tissue. X = 200 µm.

The data presented in Table 6 and Figure 4 indicate the lowest features of leaf anatomy in the common bean plant, as shown in the treatment of comparison (control; foliar spray with d.W), which reflected the negative effect of soil salinity. However, foliar nourishment with Se-NPs positively affected leaf anatomical features, as it led to significant elevations in all features of leaf anatomy compared to the comparison treatment. The best-applied rate for Se-NPs was 1 mM, which maximized the results of all features of leaf anatomy. It increased the thickness of each of blade, palisade tissue, spongy tissue, phloem, and xylem by 92.1%, 145.5%, 111.4%, 108.4%, and 100.3%, respectively, along with increasing midvein length and width by 85.2% and 107.3%, respectively. It also increased the diameter of the vessel by 118.7% compared to the comparison treatment.

## 4. Discussion

The agricultural sector in arid and semi-arid regions, including Egypt, faces many difficulties, including soil salinity. Salinity causes physiological drought even in the presence of water due to high osmotic pressure, which impedes plant growth and productivity due to disturbance of cell metabolism, division, elongation, meristematic activity, pigmentation, photosynthesis-related efficiency, stomatal closure, ionic imbalance, and gaseous exchange, along with elevated respiration rate and oxidative stress markers, including hydrogen peroxide (H_2_O_2_), superoxide (O_2_^•−^) radical, and hydroxyl anion (OH^−^), due to the failure of antioxidant defenses to withstand salt stress [14,45,46,47,48,49,50].

Plants possess an antioxidative system to mitigate the damaging effects of ROS [51]. This system contains many active ingredients with low-molecular mass (non-enzymatic antioxidants) and high-molecular mass (antioxidant enzymes) that synergistically act with osmoprotectant compounds [52,53]. However, under severe stress, the natural tolerance of plants does not help with stress resistance since the active ingredients of the plant with low-molecular mass and high-molecular mass do not provide sufficient requirements for defense against stress. Therefore, exogenous adjuvants, including beneficial nutrients in nanoparticles and other auxiliary strategies, must be used to raise the efficiency of antioxidant defense in stressed plants to enable them to function effectively under different stresses [7,10,54,55,56,57,58,59], including salinity [6,60,61,62].

In the present study, the exogenous adjuvant was selenium nanoparticles (Se-NPs) applied as a foliar spray, which enabled common bean plants to perform effectively under soil salinity conditions (EC = 7.55–7.61 dS m^−1^; Table 1). Se-NPs enhanced the growth and production of salt-stressed common bean plants by promoting the aqueous state of plant cells, delaying plant aging [63,64], and improving the size and/or the number of plant cells. These distinct results may be attributed to the maintenance of cell turgidity, leading to cell elongation [65], increased rigidity of mature leaves [66,67], and improved all photosynthesis-related attributes [68], thus promoting the efficiency of photosynthesis [69]. Therefore, Se-NPs, applied as a foliar application, could be an effective biostimulator to promote tolerability of common bean plants under conditions of salinity stress [70]. Several works have used Se-NPs to improve plant growth under various stresses in different crops such as sorghum under heat stress [71], tomato under salinity stress [72], wheat under drought stress [73], wheat under heat stress [64], and maize under salinity stress [65]. Low concentrations of Se-NPs have been used to stimulate plant growth and yield; however, high doses cause toxic effects [65,67]. In this concern, Hawrylak-Nowak [74] found that application of low concentration of Se (5 μmol·dm^−3^) stimulates plant growth and root elongation, but high concentrations (50 and 100 μmol·dm^−3^) of Se leads to decreased growth, biomass, and root tolerance index resulting from high phosphorous (P) accumulation in the straw of plants. Our resulting data demonstrate that Se-NPs applied at 1.5 mM reduced growth, yield, and all biochemical indices compared to 1.0 mM, indicating that concentrations of Se-NPs greater than 1.0 mM caused damage to salt-stressed *Phaseolus vulgaris* plants.

Oxidative stress induced by salinity causes damage to plasma membranes and chlorophyll due to an excess of H_2_O_2_ and O_2_^•−^ [15]. It is associated with raised uptake of Na^+^ and limited accumulation of Mg^2+^, which affects the synthesis of the chlorophylls [75]. Subjecting common bean plants to salinity-induced adverse conditions induces changes in the role of the complex of pigment protein [75]. Salt accumulation limits pigment synthesis by minimizing the activity of Mg-chelatase, 5-aminolevulinic acid dehydratase, porphobilinogen deaminase, protochlorophyllide oxidoreductase, and porphyrinogen IX oxidase [76]. These undistinguished findings are associated with increased activity of chlorophyllase [77], diminished leaf water potential, reduced uptake of N, and thus minimized efficiency of photosynthesis [78,79]. However, Alyemeni et al. [80] reported that Se application helps eliminate reactive oxygen species (ROS) and nutrient build-up due to improved photosynthesis. Additionally, Elkelish et al. [77] documented that exogenous Se application enhanced chlorophyll and carotenoid pigments and conferred a positive effect on stomatal conductance and photosynthetic efficiency. Besides, Iqbal et al. [64] showed that applying the optimal amount of Se leads to an increase in chlorophyll and protects the chloroplast structure from oxidative damage. The decrease in oxidative damage occurs due to the positive effect of Se on the chlorophyll content and its role in the structure of chloroplasts [65,81]. The Se-induced improvement in photosynthesis has also been reported in tomato plants that interfered with salinity [72]. Se had a considerable potency for maintaining the ultrastructure of mitochondria and chloroplasts to promote photosynthesis capacity and acclimatize to salt stress [81]. The Se-NPs exhibit a profitable influence on the performance of the photosystem in stressed plants [21,82,83].

Principally, Rios et al. [84] documented that the decrease of photosynthesis under salinity stress occurs due to oxidative and osmotic stress and nutritional imbalance. Our resulting findings reveal that foliar nourishment with 1 mM Se-NPs as an optimal level increased photosynthetic effectiveness, transpiration rate (*Tr*), net photosynthesis rate (*Pn*), and leaf gas exchange in salinity-stressed *Phaseolus vulgaris* plants (Table 4). Optimum supplementation of Se boosted the performance of photosynthesis by promoting indices of chlorophyll fluorescence, assimilation of CO_2_, and photosynthesis rate under the stress conditions of salinity [80,85]. Our data show that Se-NPs applied at 1 mM significantly enhanced cell integrity (i.e., RWC and MSI) under salt stress (Table 5) by increasing hydraulic conductivity of roots which caused an increase in the flow of water from roots to shoots of plants [86,87,88,89]. Therefore, Se-NPs application eliminates the toxic effect of salinity and increases RWC by increasing water flow from roots to shoots. The increased RWC by Se-NPs is associated with an accumulation of sugar content that reflects positively on plant productivity under stress conditions [90]. Our results also show that Se-NPs increased proline accumulation that improved the effectiveness of photosynthesis, production of ATP, and efficiency of water use by plants, as formerly reported [91]. Proline is a low-molecular-mass antioxidant that has an important role in the osmotic modification of salinity-stressed plant cells [92]. It reduces ROS damage and promotes the tolerability of plants by minimizing ROS detoxification under salt stress [93]. Besides, proline improves plant antioxidant system efficiency [94]. Likewise, Chandrasekhar and Sandhyarani [95] stated that the accumulation of proline assists plants replenish energy under salinity conditions and increases their survival under stress. Se-NPs application regulates the accumulation of proline and increases enzymatic activity and protects photosynthesis by improving RWC and Rubisco protection along with preserving proline and soluble sugar contents to help clean up ROS [78].

Similar to proline, soluble sugar accumulation keeps the regularity between the osmotic quality of the cellular cytosol and the vacuole [96]. Se appears to be able to positively modulate accumulations of soluble sugars and proline in plant leaves under salinity stress due to their significant roles in maintaining osmotic and ionic balance in plant cells, thus leading to stress tolerance [77,78]. There are additional mechanisms to minimize ROS damage and promote plant tolerance by proline. Among these mechanisms, it reduces the influences of salinity stress by cleaning up ROS generated by salinity poisoning and may react with hydroxyl radicals, directly, or quench singlet oxygen in a physical manner [93].

Our resulting data indicate the determination of the peroxidation level of membrane lipids as the content of MDA, and this is rated as a stress biochemical indicator, as it minimizes biomass production and decreases the plant’s stress adaptation [97]. Our data reveal that the control treatment (soil salinity with foliar spray with distilled water) exceeded all Se-NPs at O_2_^•−^ and H_2_O_2_ levels as markers of oxidative stress and their unwanted consequences such as increased electrolyte leakage (EL) and MDA levels (Figure 1). This realistic result was demonstrated by the high ROS concentrations generated in the control treatment plants. Commonly, the levels of peroxidation of membrane lipids and EL in higher plants through free radicals encourage damage to cell membranes or retrogradation when plants undergo ecological stresses [98]. Under these conditions, acceptance of electrons by NADP will be restricted, thus O_2_ can act as an electron acceptor, resulting in the production of more ROS, which cause peroxidation of cell membranes and increased EL [99,100]. However, the application of Se-NPs significantly controlled O_2_^•−^ and H_2_O_2_ (Figure 1) and reduced MDA and EL levels (Figure 1). Salinity stress causes oxidative cell damage through an imbalance in ROS generation and alters antioxidant activity [101]. To avert the oxidative damage caused by salt stress, plants possess a well-developed antioxidative system, with low- (non-enzymatic) and high-molecular-mass (enzymatic) active ingredients. Among the enzymes, SOD (first line of defense) defends against O_2_^•−^ [102] by converting it to H_2_O_2_, which in turn is converted to O_2_ and H_2_O by peroxidases in the presence of APX, which is the most important enzyme in reducing ROS [103,104]. GR and DHAR as regenerating enzymes have been described as a base portion of the Halliwell–Asada cycle. They form a portion of AsA regeneration from DHA using GSH as a reducing power [105]. The present study indicated that Se-NPs treatments, especially that applied at 1 mM, significantly increased antioxidant enzyme activities (e.g., CAT, APX, GR, SOD, and POX) compared to the control treatment (Figure 2). These increased enzyme activities upregulate the AsA-GSH pathway, protecting the photoelectron transport chain by respecting toxic radical formation and NADP level maintenance. Besides, applying Se reduces ROS and promotes plant growth by stimulating various antioxidants and modified osmoprotectant levels. Likewise, former studies have documented that applying Se increases the activity of antioxidant enzymes in different crops [80,81,82].

The data from our study show minimal accumulation of Na^+^ in common bean plants due to the application of Se-NPs, thus reducing levels of oxidative stress markers induced by salinity (Figure 3). Zhang et al. [106] hypothesized that the application of Se-NPs elevates the expression of Na^+^/H^+^ antiport and tonoplast H^+^-ATPase at the root membranes, limiting the translocation of Na^+^ ions to the upper plant tissues, thus limiting Na^+^ toxic effects. Our data also show that the application of Se-NPs significantly increased the uptake and thus the content of N, P, and K^+^ to promote the regulation of plant growth by inference to antioxidant metabolism, cellular stress signaling, and nitrogen assimilation [78,107,108]. Besides, high N, P, and K^+^ uptake and minimization of Na^+^ level in common bean plants lead to improved stress signaling and amino acid and metabolite production, thus boosting plant tolerance to salinity [77]. As another important mechanism, Astaneh et al. [109] stated that spraying leaves with Se leads to Na^+^ and K^+^ homeostasis under salt stress due to that Se plays a positive role at the level of membrane transport and improves expression of the *ZmNHX1* gene, thus improving K^+^ uptake and reducing Na^+^ accumulation under salt stress [17,110]. This improved ratio of K^+^/Na^+^ has been shown to be beneficial for protecting vital processes and maintaining osmotic balance [17,111]. In plant roots, the ZmMPK5, ZmMPK7, and ZmCPK11 genes are upregulated by Se under osmotic stress. Besides, overexpression and upregulation of ZmNHX1 and NHX genes in some transgenic plant species are responsible for Na^+^ compartmentalization and enhanced salt resistance [17,65,112]. In addition, OsNHX1 (vacuolar Na^+^/H^+^ antiporter gene) is responsible for maintaining plant osmotic balance by reducing Na^+^ ions obstruction during water movement towards plant shoots [17,112], which may be attributed to the sequestration of Na^+^ ions in vacuoles of roots and/or shoots [17,113]. It could be visualized that the higher OsNHX1 transcriptional level promoted Na^+^ sequestration within the root vacuoles, thus reducing Na^+^ ion accumulation in the plant shoot, which ultimately promoted plant growth and antioxidant defense mechanisms [17].

Our resulting data show an increase in endogenous Se as a result of exogenous feeding with Se-NPs, and the optimum endogenous content of Se (28.2–29.4 mg kg^−1^ DW) was obtained by exogenous application of 1 mM Se-NPs (Table 5). The promoted growth of *Phaseolus vulgaris* plants by externally used Se-NPs could be a positive measure of salt tolerance associated with increased plant productivity, and thus the plants produced more metabolites, which were essential for plant growth. Foliar nourishment with Se-NPs improved salinity stress tolerance in *Phaseolus vulgaris* plants through optimal growth and productivity due to the optimal content of endogenous Se. These desirable findings are attributed to the restoration of the damaged chloroplast structure along with stimulation of chlorophyll biosynthesis and increased photosynthetic capacity and antioxidant activities due to the osmoprotection and osmoregulation role of Se [114,115]. The osmoregulation role of Se is effectively increased through its upregulation of choline/choline monooxygenase biosynthesis, which catalyzes glycine betaine biosynthesis [116]. Thus, Se plays some crucial roles, as it helps in stabilizing and maintaining membrane integrity and keeps the turgor of plant cells under stress conditions of salinity.

Our findings also show that the application of Se-NPs improved the leaf anatomical features in salinity-stressed *Phaseolus vulgaris* plants, indicating that exogenous Se-NPs recovered the deleterious effects of salt stress on the features related to leaf anatomy. This improvement in features related to leaf anatomy awarded an opportunity for a good translocation of assimilates along with absorbed nutrients into cells for use in various metabolic processes that are reflected in a positive manner in robust growth and satisfying productions under salt stress conditions. Leafy nourishment with Se-NPs could improve the protective tissue development in *Phaseolus vulgaris* leaves, thus strengthening their anti-dehydration capacity. Similar anatomical findings have been obtained previously [3,55,117].

Under salinity stress, our resulting data display that low- and high-molecular-mass antioxidant activity was sufficiently increased by application of Se-NPs (at a concentration of 1.0 mM), in conjunction with higher RWC, MSI, stomatal conductance, and photosynthesis efficiency and decreased EL and MDA, thus reducing cell membrane injury. Finally, our data suggest that Se helps minimize salt stress influences in *Phaseolus vulgaris* plants by promoting antioxidative activity. In addition, there is evidence that the resulting yields from plants treated with Se are found to be safe for human health [118].

## 5. Conclusions

The results obtained indicate that the decreased parameters (pigments and other indices related to photosynthetic efficiency, osmoprotectants, various antioxidants, and nutrients) related to *Phaseolus vulgaris* plant growth and yield obtained under salty field conditions (EC = 7.55–7.61 dS m^−1^) were restored with nourishment with Se-NPs applied as a foliar spray for plants, especially at 1 mM. Se-NPs application ameliorated photosynthetic efficiency, antioxidant defense system, and osmoregulation and promoted plant growth and productivity. Thus, foliar nourishment with Se-NPs provided a noticeable role in alleviating the adverse effects of salt stress on all indices related to *Phaseolus vulgaris* plant growth, physiology, biochemistry, and green yield. Therefore, leafy nourishment with Se-NPs can be recommended as a noteworthy strategic approach to promote the growth and productivity of the *Phaseolus vulgaris* plant under soil salinity stress at the open field level.

## Figures and Tables

**Table 1:** Some major properties of the investigated soil.

Soil Characteristics	Unit	Values	
		First Season	Second Season
Sand	%	44.2	44.7
Silt		31.7	30.8
Clay		24.1	24.5
Texture class		Loam	
Field capacity	%	16.2	16.3
CaCO_3_	g kg^−1^	60.1	62.3
Organic matter		7.55	8.25
pH (in soil paste)		7.52	7.61
EC (in soil paste extract)	dS m^−1^	7.55	7.61
Soluble ions (anions and cations) **			
Mg^2+^	mmol_c_ L^−1^	19.9	20.1
Ca^2+^		15.6	15.7
SO_4_^2–^		8.19	8.4
K^+^		6.72	6.53
HCO_3_^−^		20.9	20.7
CO_3_^2–^		-	-
Na^+^		19.1	19.5
Cl^−^		31.7	32.7
Available nutrient			
N	mg kg^−1^ soil	59.0	59.4
P		9.80	9.90
K		98.7	99.5
Se		0.06	0.08

** Mg^2+^ = magnesium cation, Ca^2+^ = calcium cation, SO_4_^2^^−^ = sulfate anion, K^+^ = potassium cation, HCO_3_^−^ = bicarbonate anion, CO_3_^2^^−^ = carbonate anion, Na^+^= sodium cation, and Cl^−^ = chloride anion.

**Table 2:** Some major properties of the Se-NPs used in this study.

The Property	The Unit	The Value
Diameter	nm	80
Surface area	m^2^ g^−1^	30−50
Density	g cm^−3^	3.89
Purity	%	99.5
Morphology	Spherical	

**Table 3:** Growth and productivity responses of salt-stressed common bean plants (soil EC = 7.55−7.61 dS m^−1^) to foliar nourishment with selenium dioxide nanoparticles (Se-NPs) in two consecutive seasons.

Foliar Spray	ShL (cm)	NoL-P	LeA-P (cm^2^)	DW-Sh (g)	NoP-P	GPY-H (ton ha^−1^)
The first season (2019)						
Distilled water	16.1 ± 1.1 d	6.87 ± 0.4 b	7.26 ± 0.3 c	3.61 ± 0.1 d	10.6 ± 0.9 d	2.17 ± 0.19 c
Se-NPs (0.5 mM)	21.5 ± 1.4 c	8.24 ± 0.6 b	7.55 ± 0.4 c	6.17 ± 0.3 c	12.6 ± 1.1 c	4.33 ± 0.21 bc
Se-NPs (1 mM)	32.3 ± 2.1 a	10.7 ± 0.8 a	9.25 ± 0.6 a	9.31 ± 0.6 a	18.8 ± 1.5 a	7.88 ± 0.26 a
Se-NPs (1.5 mM)	26.1 ± 1.6 b	9.82 ± 0.4 a	8.65 ± 0.5 b	7.64 ± 0.4 b	14.9 ± 1.3 b	6.74 ± 0.19 ab
The second season (2020)						
Distilled water	16.9 ± 1.3 d	7.77 ± 0.3 c	7.76 ± 0.2 d	4.11 ± 0.2 c	11.1 ± 0.8 d	2.21 ± 0.12 c
Se-NPs (0.5 mM)	22.4 ± 1.5 c	9.21 ± 0.5 b	8.29 ± 0.4 c	6.39 ± 0.3 b	13.7 ± 1.2 c	4.40 ± 0.14 bc
Se-NPs (1 mM)	33.6 ± 2.5 a	12.0 ± 0.7 a	10.0 ± 0.8 a	9.39 ± 0.6 a	19.9 ± 1.2 a	7.95 ± 0.31 a
Se-NPs (1.5 mM)	27.2 ± 2.3 b	10.9 ± 0.6 a	9.35 ± 0.6 b	7.71 ± 0.5 b	15.8 ± 1.3 b	6.81 ± 0.21 ab

Data are means (n = 9) ± SE. In each column, means with different letters are considered significantly different at *p* ≤ 0.05. ShL, shoot length; NoL-P, plant leaf number; LeA-P, plant leaf area; DW-Sh, shoot dry weight; NoP-P, plant pods number; GPY-H, hectare green pods yield.

**Table 4:** Photosynthetic efficiency responses of salt-stressed common bean plants (soil EC = 7.55–7.61 dS m^−1^) to foliar nourishment with selenium dioxide nanoparticles (Se-NPs) in two consecutive seasons.

Foliar Spray	Chlorophyll “a”	Chlorophyll “b”	Carotenoids	*Pn* (µmol CO_2_ m^−2^ s^−1^)	*Tr* (mmol H_2_ O m^−2^ s^−1^)
	(mg g^−1^ FW)				
The first season (2019)					
Distilled water	0.94 ± 0.07 d	0.53 ± 0.02 c	0.85 ± 0.03 c	5.56 ± 0.2 d	3.18 ± 0.1 d
Se-NPs (0.5 mM)	1.11 ± 0.08 c	0.62 ± 0.03 b	0.98 ± 0.04 bc	9.77 ± 0.4 c	4.76 ± 0.2 c
Se-NPs (1 mM)	1.33 ± .0.06 a	0.71 ± 0.04 a	1.20 ± 0.06 a	12.9 ± 0.5 a	7.01 ± 0.5 a
Se-NPs (1.5 mM)	1.20 ± 0.08 b	0.65 ± 0.02 ab	1.07 ± 0.07 ab	11.2 ± 0.6 b	5.97 ± 0.3 b
The second season (2020)					
Distilled water	0.97 ± 0.04 c	0.55 ± 0.01 b	0.90 ± 0.02 b	5.86 ± 0.3 d	3.44 ± 0.1 d
Se-NPs (0.5 mM)	1.14 ± 0.06 b	0.65 ± 0.02 a	1.01 ± 0.04 b	9.88 ± 0.6 c	4.94 ± 0.1 c
Se-NPs (1 mM)	1.36 ± 0.07 a	0.74 ± 0.02 a	1.24 ± 0.04 a	13.0 ± 0.8 a	7.26 ± 0.4 a
Se-NPs (1.5 mM)	1.23 ± 0.05 b	0.68 ± 0.03 a	1.10 ± 0.03 ab	11.5 ± 0.7 b	6.18 ± 0.3 b

Data are means (n = 9) ± SE. In each column, means with different letters are considered significantly different at *p* ≤ 0.05. *Pn*, rate of net photosynthetic; *Tr*, rate of transpiration.

**Table 5:** Tissue cell integrity response of salt-stressed common bean plants (soil EC = 7.55–7.61 dS m^−1^) to foliar nourishment with selenium dioxide nanoparticles (Se-NPs) in two consecutive seasons.

Foliar Spray	RWC (%)	MSI (%)	Free proline (µmol g^−^^1^ DW)	Soluble sugars (mg g^−^^1^ DW)	Se content (mg kg^−1^ DW)
The first season (2019)					
Distilled water	77.1 ± 2.2 c	36.9 ± 1.2 d	27.5 ± 1.3 d	18.6 ± 1.1 d	10.4 ± 0.2 d
Se-NPs (0.5 mM)	79.6 ± 2.6 c	48.9 ± 1.5 c	29.5 ± 1.2 c	21.3 ± 1.3 c	21.6 ± 0.4 c
Se-NPs (1 mM)	90.6 ± 3.5 a	81.4 ± 2.5 a	34.1 ± 1.3 a	26.2 ± 1.4 a	28.2 ± 0.5 b
Se-NPs (1.5 mM)	86.3 ± 3.3 b	61.0 ± 1.8 b	32.4 ± 1.5 b	24.1 ± 1.5 b	36.4 ± 0.7 a
The second season (2020)					
Distilled water	79.1 ± 2.5 b	38.2 ± 1.1 d	28.1 ± 1.4 c	19.1 ± 1.2 d	12.0 ± 0.2 d
Se-NPs (0.5 mM)	80.9 ± 3.2 b	50.6 ± 1.9 c	30.1 ± 1.5 b	21.8 ± 1.3 c	24.2 ± 0.3 c
Se-NPs (1 mM)	92.5 ± 3.4 a	83.5 ± 2.9 a	34.5 ± 1.4 a	26.6 ± 1.4 a	29.4 ± 0.6 b
Se-NPs (1.5 mM)	88.1 ± 3.5 a	63.1 ± 2.3 b	33.1 ± 1.6 a	24.5 ± 1.5 b	37.0 ± 0.8 a

Data are means (n = 9) ± SE. In each column, means with different letters are considered significantly different at *p* ≤ 0.05. RWC, relative water content; MSI, membrane stability index; Se, selenium.

**Table 6:** Anatomical features responses of salt-stressed common bean leaf (soil EC = 7.55–7.61 dS m^−1^) to foliar nourishment with selenium dioxide nanoparticles (Se-NPs) in 2020 (second) season.

Foliar Spray	Blade Thic. (μm)	Palisade Thick. (μm)	Spongy Thick. (μm)	Length of Midvein (μm)	Width of Midvein (μm)	Phloem Thick. (μm)	Xylem Thick. (μm)	Diameter of Vessel (μm)
Distilled water	130.6 ± 1.1d	40.5 ± 0.9d	54.3 ± 1.3d	750.4 ± 2.6d	619.1 ± 4.1d	101.6 ± 1.1d	105.4 ± 1.2d	15.5 ± 0.4d
Se-NPs (0.5 mM)	165.5 ± 1.5c	49.2 ± 1.2c	60.6 ± 1.2c	904.5 ± 3.5c	722.2 ± 2.5c	117.3 ± 1.4c	113.32 ± 1.4c	20.3 ± 0.5c
Se-NPs (1 mM)	250.8 ± 2.2a	99.5 ± 1.3a	114.8 ± 2.2a	1389.9 ± 2.9a	1283.6 ± 4.3a	211.8 ± 1.5a	211.2 ± 1.9a	33.9 ± 0.8a
Se-NPs (1.5 mM)	230.3 ± 1.9b	85.4 ± 1.1b	100.6 ± 2.5b	1270.6 ± 3.5b	1172.7 ± 3.6b	181.2 ± 1.6b	145.6 ± 1.7b	29.2 ± 0.7b

Data are means (n = 9) ± SE. In each column, means with different letters are considered significantly different at *p* ≤ 0.05. Thick., thickness.

## Data Availability

The data presented in this study are available upon request from the corresponding author.
