# Correction: Rady et al. Foliar Nourishment with Nano-Selenium Dioxide Promotes Physiology, Biochemistry, Antioxidant Defenses, and Salt Tolerance in *Phaseolus vulgaris*. *Plants* 2021, *10*, 1189

**DOI:** 10.3390/plants14162455

**Published:** 2025-08-08

**Authors:** Mostafa M. Rady, El-Sayed M. Desoky, Safia M. Ahmed, Ali A. Majrashi, Esmat F. Ali, Safaa M. A. I. Arnaout, Eman Selem

**Affiliations:** 1 Botany Department, Faculty of Agriculture, Fayoum University, Fayoum 63514, Egypt; sma20@fayoum.edu.eg; 2 Botany Department, Faculty of Agriculture, Zagazig University, Zagazig 44519, Egypt; sayed1981@zu.edu.eg (E.-S.M.D.); sea_sound16@zu.edu.eg (S.M.A.I.A.); 3 Department of Biology, College of Science, Taif University, P.O. Box 11099, Taif 21944, Saudi Arabia; aa.majrashi@tu.edu.sa (A.A.M.); a.esmat@tu.edu.sa (E.F.A.); 4 Botany and Microbiology Department, Faculty of Science, Zagazig University, Zagazig 44519, Egypt; eman8_extra8@yahoo.com

## Error in Author Name

It should be Ali A. Majrashi, not Ali Majrashi. 

## Error in Abstract

Se-NPS should be Se-NPs.

## Error in Table 2

In the original publication [1], there was a mistake and the corrections are as follows:

### 2.2. Foliar Application of Nano-Selenium (Se-NPs)

The Se-NPs was purchased from Sigma Aldrich Co. (Sigma-Aldrich, 99.5% purity of Se-NPs) with properties shown in Table 2.

**Table 2:** Some major properties of the Se-NPs used in this study.

The Property	The Unit	The Value
Diameter	nm	80
Surface area	m^2^ g^−1^	30−50
Density	g cm^−3^	3.89
Purity	%	99.5
Morphology	Spherical	

The authors state that the scientific conclusions are unaffected. This correction was approved by the Academic Editor. The original publication has also been updated.
